# Prescriptions

**Published:** 1893-04

**Authors:** 


					﻿Prescription Department.
whooping Cough.
Dr. Farquahar, of Zanesville, Ohio,
praises the action of:—
B Croton chloral........gr. xv.
Etheris..........    .gtt.	xx.
Pot. brom..........3j.
Tr. bellad.........gtt. xv.
Tr. hyoscyam.....gtt. xxiv.
Syr. tolutan. q. s. adf § iv.
M. Sig.—A teaspoonful four times
a day.
COUGH FIXTURE FOR INFANTS.
B Tinct. Opii Camphoratae._ 1 oz.
Spts. Ammo. Aromatici.. 1 oz.
Syr. Pruni Virginianse... 1 oz.
Ext. Ipecac..................30	m.
Aquae.......q. s.ad..... 8 oz.
M. Sig.—A teaspoonful
— The Prescription.
SORE THROAT.
The following gargle for sore throat
is given Les Nouveaux Remedies:—
B Crystallized carbolic acid. 3 ss.
Absolute alcohol........3 ij.
Oil of peppermint.......gtt. x.
NOCTURNAL EMISSIONS.
B Ext-, aloes.........gr.	iij.
Ext. belladonna.. ,gr. ij.
Ext. hyoscyami.. ?gr. iv.
Pulv. camphorae.. .gr. xxiv.
M, Ft. pilulae No. xii. Sig.—One.
pill every night.—Ex,
VOMITING IN PREGNANCY.
B Tinct. Iodi.. . . . )	,
Chloroformi.... y eQnal parts.
M. Sig.—Take five drops in a little
water, at meal time, morning and even-
ing.—Cincin. Med. News.
SORE THROAT.
R	Listerine...........jij.
Tannic acid..........Qj.
Glycerine............§j.
Water................
M. Sig. — Uuse as a gargle every
three to four hours.
WHOOPING COUGH.
R	Antipyrine........3 iss.
Potass, bromidi..3 iiss.
Elix. simp.........§ ’v-
M.	Sig. — Teaspoonful every three
hours. — H. F: Baoivnlee, M. D.,
Danbnry, Ct.
WHOOPING COUGH.
R Vini. antimonii........
Syr, scillse...aa.... 3 iij.
Aluminii ............3 ij.
M. Sig.—Teaspoonful every two or
three hours.
DIFFICULT URINATION AFTER CONFINE-
MENT.
R Tinct. Cantharidis.. .3 drachms.
Tinct. NucisVomicse.5 drachms.
M. Sig.—Eight drops every four
hours in one ounce of cold inftision of
parsley root.—Med. Snm.
INCONTINENCE OF URINE.
R Tinct Nucis Vomicae.6 drachms.
Ext. Damianae fl.... ^Jounces.
Glycerini.. q. s. ad.. 4 ounces.
M. Sig.—One drachm three times a
day, after meals, in a wineglassful of
water.
ACUTE CATARRH.
R Tr. iodine............5SS«
Acid carbolic.......3j.
M. Sig. —Place a small wide mouthed
bottle containing a moistened sponge
in a vessel of hot water. Drop five or
ten drops of the solution on the sponge
and as the vapor of the iodine ascends
with the vapor of the water, inhale it.
—Bartholow.
				

